# BMP signaling modulation attenuates cerebral arteriovenous malformation formation in a vertebrate model

**DOI:** 10.1038/jcbfm.2014.134

**Published:** 2014-07-23

**Authors:** Brian P Walcott

**Affiliations:** 1 Department of Neurosurgery, Massachusetts General Hospital and Harvard Medical School, Boston, Massachusetts, USA; 2 Cardiovascular Research Center, Massachusetts General Hospital and Harvard Medical School, Charlestown, Massachusetts, USA

**Keywords:** arteriovenous malformation, losartan, medical therapy, stroke, zebrafish

## Abstract

Cerebral arteriovenous malformations (AVMs) are vascular anomalies that carry a high risk of stroke and death. To test potential AVM therapies, a reverse genetics approach was used to model AVMs in zebrafish. Antisense morpholino oligonucleotides were used to knockdown *activin receptor-like kinase I* (*alk1*), which encodes a transforming growth factor (TGF)-beta family type I receptor implicated in a subset of human AVMs. Knockdown of *alk1* caused a spectrum of morphologic, functional, and molecular defects that resemble those seen in humans with AVMs. It was found that losartan, an angiotensin II receptor antagonist, attenuated abnormal blood vessel morphology and systemic manifestations of high-output arteriovenous shunting *in vivo.* SMAD1 phosphorylation was significantly decreased in *alk1* morphants compared with uninjected organisms (0.189±0.0201, 0.429±0.0164, *P*=0.0002). After treatment, morphant SMAD1 levels approached uninjected levels (0.326±0.0360, *P*=0.0355) and were significantly higher than those seen in the morphant-control group (*P*=0.0294). These data suggest that modulating the BMP signaling pathway with losartan, a drug in widespread clinical use in humans as an antihypertensive, may have the potential to be further evaluated as a therapeutic strategy for patients with AVMs.

## Introduction

Arteriovenous malformations (AVMs) of the brain are vascular anomalies of children and adults that carry a high risk of stroke.^1^ Their primary pathologic phenotypic characteristic is a direct communication between arteries and veins without an intervening capillary bed. Treatment of these lesions is limited to surgery, focused external beam radiation, and/or endovascular embolization; no specific FDA (Federal Drug Administration)-approved pharmacologic therapies currently exist. For unruptured cerebral AVMs, a recent randomized trial of these interventional procedures versus conservative management (A Randomized Trial of Unruptured Brain Arteriovenous Malformations, ‘ARUBA'—ClinicalTrials.gov identifier NCT00389181) was undertaken to determine the best management paradigm. In 2013, the National Institute of Neurological Disorders and Stroke halted randomization and enrollment within the ARUBA trial, underscoring the procedural risk associated with treatment of cerebral AVMs with currently available modalities and the need for new treatments.

The process by which AVMs develop is poorly understood, although aberrations in blood vessel formation and segregation during embryonic development and adult life are thought to be responsible. In development, the establishment of a vascular identity (be it arterial or venous) initiates from molecular signals that result in functional, and subsequently structural changes.^2, 3^ There are several hierarchical signaling pathways that promote or inhibit divergent endothelial cell fates, including hedgehog,^4^ vascular endothelial growth factor,^5, 6^ notch,^7, 8^ transforming growth factor (TGF)-beta,^9^ and the ephrin ligand-receptor pathway,^10^ among others. These pathways have a complex interaction and are crucial regulators of vascular assembly, differentiation, and boundary formation.

Despite a robust understanding of vasculogenesis and angiogenesis, the mechanisms behind the formation of discrete AVMs are not well known. These lesions occur either sporadically or much more rarely, in the context of a hereditary syndrome. One such syndrome in humans, hereditary hemorrhagic telangiectasia type-2, is caused by a mutation in the gene encoding activin receptor like kinase 1 (*ALK1* or *ACVRL1*), a type I TGF-beta receptor in the BMP signaling pathway.^11^ This known mutation provides an opportunity to study the pathogenic mechanisms of AVMs in vertebrate models. The optical clarity of zebrafish embryos with fluorescent vasculature, their ability to be genetically altered with knockdown technology, and their ability to be treated with small molecules permits the study of cerebral AVMs *in vivo* on an unprecedented scale.^12^ This zebrafish model was utilized in experiments with the goal of identifying a small molecule with activity against cerebral AVM formation.

## Materials and methods

The investigation was approved by the Massachusetts General Hospital Institutional Review Board and conforms to the ‘Guide for the Care and Use of Laboratory Animals' published by the US National Institutes of Health (NIH Publication No. 85-23, revised 1996).

### Morpholino Injection

A custom, *alk1* splice-site blocking morpholino (5′-ATCGGTTTCACTCACCAACACACTC-3′) was generated (Gene Tools, LLC, Philomath, OR, USA). This morpholino has previously been shown to generate AVMs in zebrafish.^13^ Morpholino efficacy was previously verified by PCR identification of splicing defects in the targeted genes. The morpholino was resuspended in 1 × Danieu's solution and 2.5 ng was injected into the cell bodies of fertilized eggs from *Tg(flk1:EGFP)* zebrafish pair matings at the single-cell stage. Arteriovenous malformations were generated in >98% of organisms.

### Zebrafish Maintenance and Drug Exposure

Zebrafish were raised in accordance with standard husbandry protocols. (The age of embryos is indicated by the hours after fertilization and days after fertilization for all experimental data shown.) Morpholino-injected or -uninjected embryos were placed into a 96-well microtiter plate with buffered embryo water (E3). The total volume of the embryos and the E3 was 100 *μ*L per well. These plates were then incubated at 28°C in the dark. All chemicals were then applied at 24 h.p.f.; losartan was used at a concentration of 20 *μ*mol/L or 100 *μ*mol/L (DMSO as a dilutant) and DMSO was used as a control, always at a concentration of <1%. The drug concentrations were determined based on preliminary experiments (dose escalation trials not shown) to identify biologically active doses that did not impair normal developmental milestones in the fish.

### Morphometric Analysis

Embryos at 4 d.p.f. were anesthetized by adding Tricaine (0.02% w/v) to E3 solution. They were then transferred to a petri dish, oriented in a sagittal profile, and examined under a Zeiss Examiner A1 stereomicroscope (Carl Zeiss Microscopy, Thornwood, NY, USA) Images were captured and then analyzed using ImageJ version 1.46 (NIH, Bethesda, MD, USA). A straight line was generated from the center of the lens to the most posterior aspect of the yolk sac. A separate straight line was generated from the posterior aspect of the yolk sac to the tip of the tail. The angle between these lines was automatically calculated, yielding a continuous variable that represented the systemic manifestations of the AVM on whole organism morphology. It is suspected that paucity of flow to the body impairs normal developmental processes, particularly with respect to renal organogenesis, resulting in organism curvature. Sixteen organisms were measured for each group (uninjected, morphant-control, and morphant-treatment).

### Fluorescent Histology

JB-4 plastic resin was selected to examine the cranial vasculature given its high level of tissue preservation, fluorescence preservation, and cellular resolution. In all, 4 d.p.f. embryos were embedded, serially sectioned, and visualized using a modification of methods previously described.^14^ The embryos were fixed in a 4% paraformaldehyde solution at 4°C overnight. They were then dehydrated in EtOH and infiltrated overnight. Embryos were then transferred to molds where embedding solution was applied. Before solidification, organisms were oriented in the same direction, parallel to the floor. Resin blocks were allowed to harden in the dark at 4°C to preserve fluorescence. A glass microtome was used to section 8 *μ*mol/L thick slices through the entire organism in the coronal plane. Samples were transferred to glass slides, where Permafluor aqueous mounting medium (Thermo Scientific, Waltham, MA, USA) and a glass coverslip were applied. Images at the level of the mid-eye (lens) were selected and the diameter of the primordial hindbrain channels (left and right) were measured using ImageJ version 1.46 (NIH, Bethesda, MD, USA). Twelve organisms were measured for each group (uninjected, morphant-control, and morphant-treatment).

### Phosphorylated SMAD1 Enzyme-Linked Immunosorbent Assay

The InstantOne ELISA kit (eBioscience, San Diego, CA, USA) was used to measure levels of phosphorylated SMAD1 (Ser463/465) in whole embryo lysates. This kit is also predicted to detect the analogous phosphorylation sites of SMAD5 and SMAD8. Using a modified protocol, six organisms per well were lysed at 4 dpf with a combination of cell lysis buffer and mechanical homogenization. Lysate and phospho-SMAD1 (Ser463/465) capture and detection antibody reagents were added simultaneously to the InstantOne assay plate. After 1 hour of incubation, wells were washed and a detection solution was applied. Absorbance was measured at 450 nm. Positive control cell lysate and negative control (cell lysis buffer) confirmed antibody efficacy. Twenty-four organisms were measured for each group (uninjected, morphant-control, and morphant-treatment). Experiments were repeated in triplicate.

### Statistical Methods

To compare continuous variables, a two-tailed Student's *t*-test was used (GraphPad, La Jolla, CA, USA). Significance was predefined at *P*<0.05. Unless otherwise stated, data are expressed as mean±s.e.m.

## Results

### Morpholino Knockdown of *alk1* Results in Cerebral Arteriovenous Malformation Formation in Zebrafish

**Figure 1:**
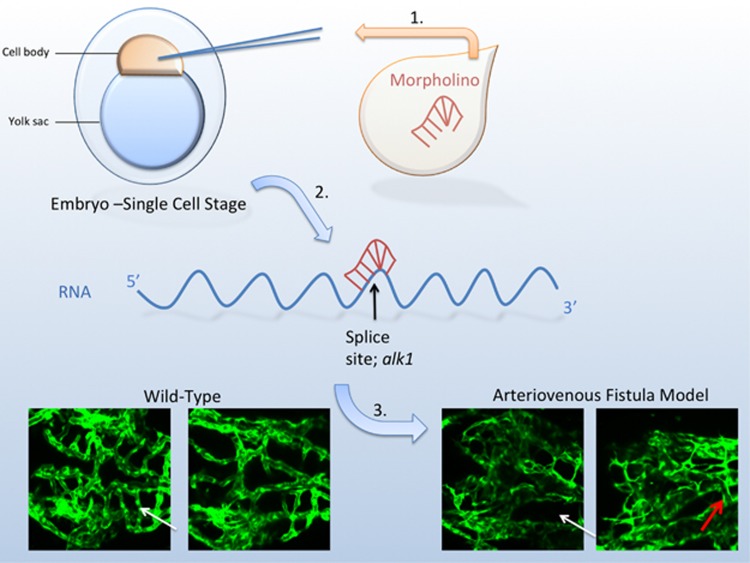
Experimental design and arteriovenous malformation (AVM) characteristics. (1) An *alk1* splice-site blocking morpholino (2.5 ng) was injected into the cell bodies of fertilized eggs from *Tg(flk1:EGFP)* zebrafish pair matings at the single-cell stage. (2) The morpholino binds to RNA, preventing translation. (3) AVMs were generated in >98% of organisms after injection. When compared with wild-type embryos, many of the connections between the basal communicating artery and the primordial midbrain channel in the morphant are not present (white arrows), analogous to the absence of an intervening capillary bed in the human condition. When a connection is present between the basal communicating artery and the primordial midbrain channel, it is an abnormal fistulous connection (red arrow). Zebrafish images are two-dimensional confocal projections of 72 hours after fertilization *Tg(flk1:EGFP)* embryos, dorsal views, anterior leftwards.

**Figure 2:**
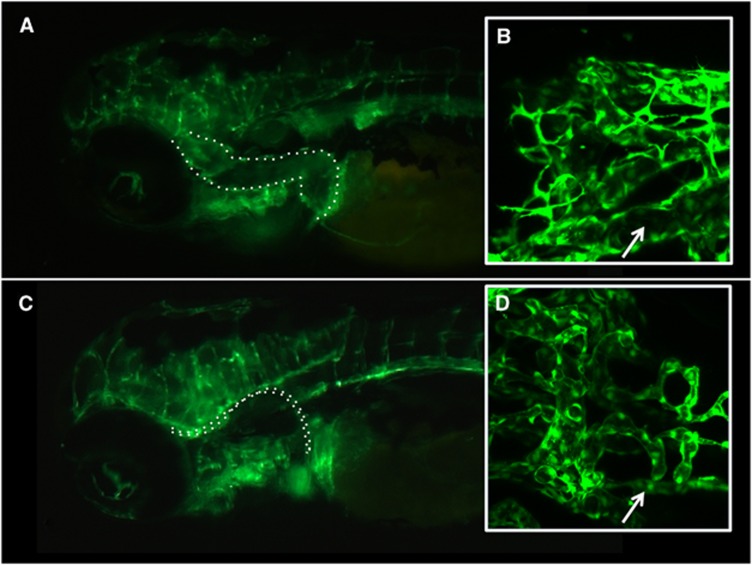
Epifluorescence and confocal microscopy of *alk1* morphants shows dilation of cranial vessels. (**A**) Morphant-control and (**C**) uninjected zebrafish at 4 dpf with endothelial expression of enhanced green fluorescent protein show that primordial hindbrain channels (outlined in white dots) are dilated in the morphant-control group compared with uninjected organisms. This channel is one of the main vessels in the cranial arteriovenous shunting circuit. Inset boxes show representative two-dimensional confocal projections of (**B**) morphant-control and (**D**) morphant-treatment zebrafish, where the outlined vessel (primordial hindbrain channel) is identified by white arrows (dorsal views, anterior leftwards). The diameter of the primordial hindbrain channel is markedly reduced in the group treated with losartan (**D**).

The genetic basis for AVMs is not well understood, making evaluation of targeted therapies challenging. However, mutations in *alk1* are associated with the development of hereditary hemorrhagic telangiectasia type-2 (OMIM 601284), a syndrome typified by the development of AVMs in numerous locations. Injection of the *alk1* morpholino in the cell body at the single-cell zebrafish embryo (Figure 1) resulted in the subsequent development of AVMs in >98% of organisms (Figure 2). Abnormal shunting of circulation was visible by 24 h.p.f., with a prominent circuit of cranial circulation. There was a paucity of circulation caudal to the heart, despite the development of normal vascular architecture. By 3 to 4 d.p.f., *alk1* knockdown zebrafish began to develop objective signs of the sequelae resulting from high-output heart failure including pericardial edema, cerebral edema, and edema surrounding the remaining yolk sac.

### Compound Selection

Losartan was selected for testing in the zebrafish AVM model given its demonstrated biologic activity seen in various aspects of TGF-beta superfamily pathways in humans. It is a widely used, orally administered FDA-approved medication for hypertension with an excellent safety profile.^15^ Although its primary target is the angiotensin II type 1 receptor (AT1), it is also known to indirectly attenuate canonical TGF-beta signaling in the vasculature.^16^ Additionally, its vascular modifying effects are also the current focus of a phase 3 clinical trial (ClinicalTrials.gov identifier: NCT00763893; Efficacy of Losartan on Aortic Dilatation in Patients with Marfan Syndrome).

### Treatment with Losartan Attenuates Abnormal Vasculature in Zebrafish Cerebral Arteriovenous Malformation Morphants

**Figure 3:**
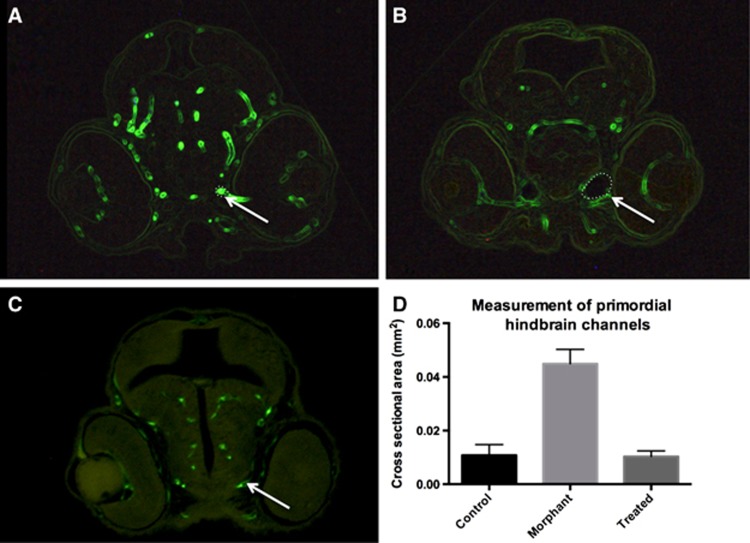
Histologic assessment of cerebral vasculature shows dilated cranial blood vessels in an arteriovenous malformation (AVM) model. 4-dpf zebrafish were embedded in JB-4 resin, serially sectioned, and visualized at the level of the mid-eye in the coronal plane. Measurements of the primordial hindbrain channels were significantly smaller in the (**A**) uninjected organisms compared with the (**B**) morphant-control group (*P*<0.0001). (**C**, **D**) Morphant-treatment group measurements (0.0103±0.00101 mm^2^) were not significantly different from uninjected organisms (*P*=0.8574), consistent with a rescue phenomenon of the vascular phenotype.

In *alk* morphants, treatment with losartan partially rescued the AVM phenotype. Measurements of the primordial hindbrain channel were significantly different between uninjected and morphant-control groups (0.0108±0.00198 mm^2^, 0.0449±0.00269, *P*<0.0001), whereas measurements from losartan-treated morphants (0.0103±0.00101) were not significantly different from uninjected organisms (*P*=0.8574) (Figure 3). Losartan treatment of wild-type organisms did not alter normal vascular development (not shown).

### Treatment with Losartan Attenuates the Systemic Organism-Level Phenotype in Zebrafish Cerebral Arteriovenous Malformation Morphants

**Figure 4:**
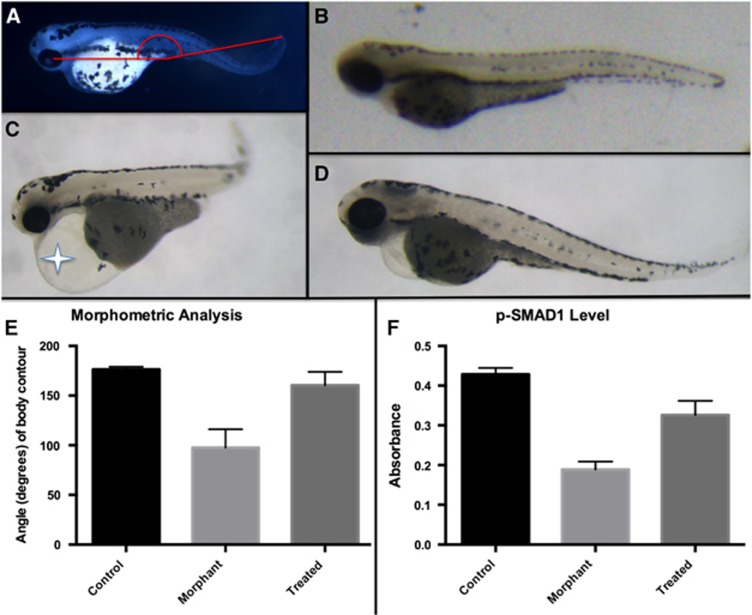
Morphometric and suppression of canonical transforming growth factor (TGF)-beta signaling changes in *alk1* knockdown zebrafish are corrected with losartan treatment. (**A**) 4 dpf zebrafish were analyzed for whole organism morphometric changes (the cranial–caudal angle) as a surrogate for pertubations in hemodynamics between groups. (**B**) In uninjected organisms, the cranial–caudal angle was measured to be 176.0±0.6488 degrees, (**C**) whereas it was significantly more acute in the morphant-control group 97.50±4.987, *P*<0.0001. Morphant organisms frequently showed systemic evidence of high-output cardiac failure, including pericardial edema (star). (**D**, **E**) Treatment with losartan increased the cranial–caudal angle to 160±3.38, which was greater than the morphant-control group, *P*<0.0001. (**F**) The InstantOne ELISA kit (eBioscience) was used to measure levels of phosphorylated SMAD1 (Ser463/465) in whole embryo lysates. Absorbance in morphant-control group was significantly decreased compared with uninjected organisms (0.189±0.0201, 0.429±0.0164, *P*=0.0002). After treatment with losartan, morphant levels approached uninjected levels (0.326±0.0360, *P*=0.0355) and was significantly higher than the morphant-control group (*P*=0.0294).

In addition to vessel architecture, the hemodynamic properties of experimental organisms were measured, using whole organism morphometric characteristics as a surrogate. In uninjected organisms the cranial–caudal angle was measured to be 176.0±0.6488 degrees, whereas it was significantly more acute in the morphant-control group 97.50±4.987, *P*<0.0001 reflecting a dorsal curvature of the tail (Figure 4) Treatment with losartan in the morphant-treatment group rescued this curvature, increasing the angle to 160±3.38, *P*<0.0001.

Functionally, hemodynamics were directly observed between the three experimental groups using light microscopy. The qualitative absence (or near absence) of blood flow in the body and tail region of any organism was identified corresponding to the *alk1* phenotype. In uninjected organisms (*n*=47), 100% showed robust body and tail circulation (Supplementary Video 1). In the morphant-control group (*n*=19), 15.8% were observed to have tail flow, compared with the morphant-treatment groups (20  *μ*mol/L losartan, 25.9% (*n*=27); 100 *μ*mol/L losartan, 40.0% (*n*=25)) (Supplementary Videos 2–4).

### Treatment with Losartan Partially Normalizes Phospho-SMAD1 Levels Caused by *alk1* Knockdown

It was hypothesized that the ability of losartan to rescue the vascular and morphologic effects of *alk1* knockdown was due to an increase in BMP pathway signaling. To test this possibility, whole embryo lysate at 4 d.p.f. was analyzed via enzyme-linked immunosorbent assay for expression of phosphorylated levels of SMAD1. SMAD1 phosphorylation was significantly decreased in *alk1* morphants compared with uninjected organisms (0.189±0.0201, 0.429±0.0164, *P*=0.0002). After treatment with losartan, morphant SMAD1 levels approached uninjected levels (0.326±0.0360, *P*=0.0355) and were significantly higher than those seen in the morphant-control group (*P*=0.0294) (Figure 4). Therefore, losartan's ability to attenuate the *alk1* knockdown phenotype may be associated with its ability to increase SMAD1 phosphorylation.

## Discussion

Currently, there are no FDA-approved pharmacologic treatments for cerebral AVMs in humans. One attractive approach for developing such therapies is repurposing of existing drugs, which could theoretically reduce the developmental and regulatory burden required for new drug approval. Therefore, an AVM model that allows rapid testing of drug candidates could be useful for identifying existing drugs with activity against AVMs.

Several mammalian models of cerebral AVMs exist, but are limited in their applicability for drug discovery. For example, cerebral AVMs have been created in swine, but this method requires a combination of surgical intervention and hemodynamic response to generate a lesion that mimics the human condition.^17^ Additionally, mice have been used as model organisms, although they require angiogenic stimulation with vascular endothelial growth factor, in addition to genetic manipulation of *alk1* to generate lesions.^18^ This confounds the interpretation of treatments such as vascular endothelial growth factor antagonists, particularly since vascular endothelial growth factor stimulation is necessary to generate the model.^19^ Zebrafish are an attractive model in that their vasculature is visualized easily with fluorescent proteins and their entire cranial circulation can be observed *in vivo*.

By using morpholino technology, we were able to manipulate the gene expression of *alk1*, creating zebrafish with cerebral AVMs.^20^ This established zebrafish AVM model recapitulates the human disease with a high level of fidelity.^13, 20^ The appearance of abnormal anomalous vessels connecting the arterial and venous circulation is *sine qua non* for the condition in humans.^21^ Beyond the appearance of the abnormal blood vessels, *alk1* knockdown zebrafish also show characteristic systemic manifestations of the accompanying pathophysiologic hemodynamic response that is seen in humans, such as in high-output cardiac failure in the pediatric population.^22, 23^

Targeted evaluation of losartan, an FDA-approved antihypertensive medication in widespread clinical use, resulted in a modest attenuation of the zebrafish AVM phenotype. This could be seen in a trend toward normalization of the abnormal cranial vasculature and hemodynamic pertubations seen with the experimental model. Furthermore, the loss of SMAD1 phosphorylation associated with *alk1* knockdown was rescued by losartan treatment.

The effects of losartan on zebrafish AVMs are not the first example of its beneficial effects on a congenital vascular condition. Losartan has been shown to prevent aortic aneurysm development^24^ and progression^25^ in a mouse model of Marfan syndrome. On the basis of these and other data, several active clinical trials are now investigating the efficacy of losartan in treating aortic aneurysms in humans. The central hypothesis underlying these trials is that losartan antagonizes TGF-beta signaling,^26, 27^ likely by inhibition of the AT1 receptor or other pathways independent of a TGF-beta receptor itself.^16^ However, less is known about the influence of AT1 on the opposing BMP signaling pathway (the pathway directly influenced by mutations in *alk1*).

**Figure 5:**
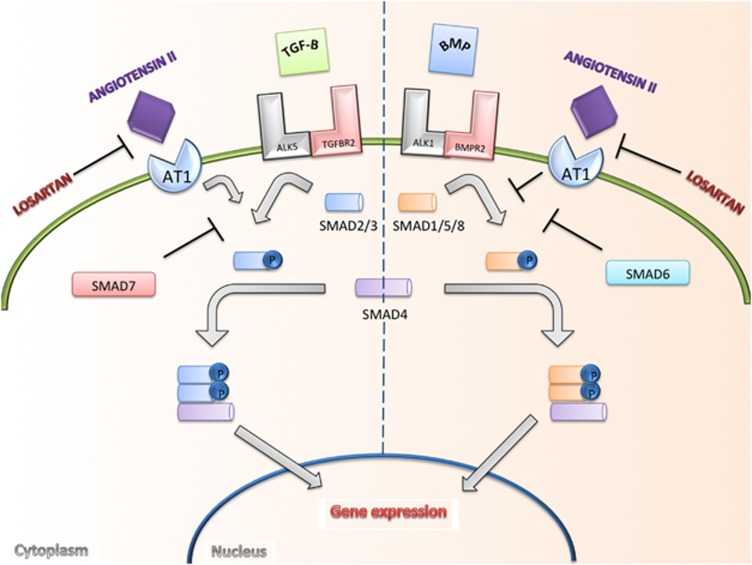
Transforming growth factor (TGF)-beta signaling pathway. Canonical signaling by TGF-beta is typically thought of in terms of two opposing, but interconnected, signaling pathways. Ligands in the TGF-beta or BMP family bind to specific Ser/Thr kinase type I (such as ALK1 and ALK5) and type II receptors (such as TGFBR2 and BMPR2) on the cell surface. Activation leads to phosphorylation of either SMAD2/3 or SMAD1/5/8, the intracellular effectors of the TGF family. These activated SMADs form complexes with SMAD4 that accumulate in the nucleus, where they go on to regulate the expression of target genes.^31, 32^ We propose that losartan has an effect on the canonical signaling cascade, independent of type I and type II TGF-beta receptors. It has been previously shown that angiotensin II type 1 receptor (AT1) blockade antagonizes AT1 receptor stimulation of TGF-beta, decreasing activation of SMAD2.^16^ The presented data suggest that activation of SMAD1/5/8 occurs after antagonism of the AT1 receptor with the use of losartan.

It was suspected that the molecular mechanism underlying the rescue mechanism of losartan treatment is related to restoration of BMP signaling. Conceptually, the TGF-beta and BMP pathways balance each other through opposing mechanisms to regulate gene expression (Figure 5).^28, 29^ *Alk1* activation normally induces phosphorylation of SMAD1/5/8,^30^ congruent with the finding that phosphorylated levels of SMAD1 were decreased in *alk1* knockdown organisms. Treatment with losartan increased the levels of phosphorylated SMAD1 in knockdown organisms, suggesting that phosphorylation of SMAD1/5/8 is necessary for transcription of specific target genes needed to maintain the differentiation between arterial and venous structures.^31, 32^ It is well known from preclinical^33, 34^ and human^35^ studies that systemic antagonism of TGF-beta signaling by losartan is possible. It is also known that levels of phosphorylated SMAD 2/3 in the TGF-beta pathway are decreased by losartan.^33, 34^ The presented data support an additional possible mechanism of action for losartan in regulating vascular development and homeostasis: activation of the BMP signaling pathway via AT1 inhibition. Alternative explanations for our findings resulting from losartan administration center around the promiscuous nature of ALK1 utilization. For example, in endothelial cells, ALK1 can partner with TGF-beta II receptors (RII) and support TGF-beta mediated SMAD1/5 phosphorylation. This same receptor complex can inhibit TGF-beta mediated SMAD2/3 phosphorylation via RII/ALK5 complex.^36^ Thus, it is also plausible that loss of ALK1 signaling leads to excessive TGF-beta mediated Smad2/3 phosphorylation in the morphant group. Further work to characterize the molecular landscape in human AVM tissue is necessary for a more complete understanding of the pathogenic mechanisms involved.

If losartan treatment is able to counteract loss of *alk1* function, how broadly might it be effective for treating AVMs? Mendelian inheritance of *alk1* mutations accounts for only a small percentage of all AVMs.^37, 38, 39^ Nevertheless, evidence exists that single-nucleotide polymorphisms in *alk1* occur in many sporadic occurring AVMs.^40, 41^ Therefore, these findings may be applicable to AVMs that occur in the sporadic setting, as well as in cases of Mendelian *alk1* mutation. Ultimately, the efficacy of any treatment must also be evaluated for the ability to cause regression of well-established lesions, which is the clinical condition seen in humans. Given losartan's ease of administration, tolerability, and favorable safety profile, future human studies of losartan may be warranted.

## Supplementary Material

Supplementary Video 4

Supplementary Videos
