# Supplementary material for: BMP signaling modulation attenuates cerebral arteriovenous malformation formation in a vertebrate model
Source: J Cereb Blood Flow Metab. 2014 Jul 23;34(10):1688–94. doi: 10.1038/jcbfm.2014.134 (PMC4269730; doi:10.1038/jcbfm.2014.134)
Supplement: Supplementary Videos [file jcbfm2014134x5.doc]

**Supplemental**

1. Video - Circulation patterns in control (wildtype) zebrafish

Circulation is visible in the transparent zebrafish without the need for mircoangiography, as seen in the tail region of a control organism. Robust arterial circulation from the dorsal aorta returns to the heart via the posterior cardinal vein.

2. Video – Magnified cranial view of an experimental model of arteriovenous malformation in *alk1* morphant zebrafish

Under light microscopy, circulatory patterns in *alk1* morphants can be seen *in vivo,* with abnormal shunting of predominantly cranial circulation.

3. Video- Whole organism view of an experimental model of arteriovenous malformation in *alk1* morphant zebrafish

In this model organism, there is a paucity of circulation caudal to the heart, despite the development of normal vascular architecture.

4. Video - Whole organism view of an experimental model of arteriovenous malformation in *alk1* morphant zebrafish treated with losartan

Treatment with losartan normalizes circulatory patterns, attenuating arteriovenous shunting in the cranial circulation and restoring blood flow to the tail.
